# DRESS syndrome induced by piperacillin-tazobactam

**DOI:** 10.1186/2045-7022-4-S3-P96

**Published:** 2014-07-18

**Authors:** Maria-Jose Sanchez-Gonzalez, Jose Barbarroja-Escudero, Dario Antolin-Amerigo, Teresa Bellon-Heredia, Victoria Lerma-Hambleton, Mercedes Rodriguez-Rodriguez

**Affiliations:** 1 Principe De Asturias University Hospital, Spain; 2 Principe De Asturias University Hospital, Allergy Department, Spain; 3 La Paz University Hospital, Investigacion Sanitaria, Spain; 4 Principe De Asturias University Hospital, Fundacion Biomedica, Spain

## Background

DRESS (drug rash with eosinophilia and systemic symptoms) syndrome is quite difficult to diagnose due to its mimic nature and an absence of a specific diagnostic confirmation technique. In fact, this entity used to be misdiagnosed. Different drug families have been described as causative agents, being the aromatic anticonvulsants the most frequent drugs involved, although other drugs as allopurinol or sulfonamides must be kept in mind too. Until now, piperacillin-tazobactam has been scarcely reported as the culprit drug.

## Method

A 38-year old man with Von Hippel-Lindau disease, with tumors resections in brain and kidney, renal insufficiency and cerebral and peritoneal metastasis, is presented. He started receiving treatment with sunitinib for his disease. He also took piperacillin-tazobactam due to a cholangitis episode. Eight days after stopping sunitinib and the 8th day of treatment with piperacillin-tazobactam, he developed persistence of fever (40ºC), generalized maculopapular erythema (but palms and soles) and pruritus, swollen face, conjunctival icterus, and laterocervical and inguinal lymphadenopathies. The blood tests revealed neutrophilic leucopenia, an increase of his basal creatinin and elevated liver transaminase levels. The following days eosinophils level reached a 14.9%.

## Results

Epicutaneous test with sunitinib 12.5 mg at 20% in petrolatum was negative at 48 and 96 hours. Epicutaneous tests with piperacillin-tazobactam at 10% in petrolatum were positive at both 48 and 96 hours. The lymphocyte transformation test (LTT) showed a negative result for sunitinib. One control subject showed also negative results. Nevertheless, this test was positive for piperacillin-tazobactam with a stimulation index over 2 in several concentrations. The patient tolerated sunitinib afterwards.

## Conclusion

In this patient, the diagnosis of DRESS induced by piperacillin-tazobactam was performed with patch tests alike with LTT.

